# Not Only Editing: A Cas-Cade of CRISPR/Cas-Based Tools for Functional Genomics in Plants and Animals

**DOI:** 10.3390/ijms25063271

**Published:** 2024-03-13

**Authors:** Aurélien Devillars, Gabriele Magon, Carlotta Pirrello, Fabio Palumbo, Silvia Farinati, Gianni Barcaccia, Margherita Lucchin, Alessandro Vannozzi

**Affiliations:** 1 Department of Agronomy, Food, Natural Resources, Animals and Environment (DAFNAE), University of Padova, Agripolis, Viale dell’Università 16, 35020 Legnaro, PD, Italy; aurelien.devillars@phd.unipd.it (A.D.); gabriele.magon@unipd.it (G.M.); fabio.palumbo@unipd.it (F.P.); silvia.farinati@unipd.it (S.F.); gianni.barcaccia@unipd.it (G.B.); margherita.lucchin@unipd.it (M.L.); 2 Research and Innovation Centre, Fondazione Edmund Mach, Via E. Mach 1, 38098 San Michele all’Adige, TN, Italy; carlotta.pirrello@fmach.it

**Keywords:** CRISPR/Cas, imaging, gene regulation, NGS, viruses, CRISPR/Cas9, CRISPR/Cas13, dCas9

## Abstract

The advent of CRISPR/Cas9 technology has revolutionized genome editing, enabling the attainment of once-unimaginable goals. CRISPR/Cas’s groundbreaking attributes lie in its simplicity, versatility, universality, and independence from customized DNA-protein systems, erasing the need for specialized expertise and broadening its scope of applications. It is therefore more and more used for genome modification including the generation of mutants. Beyond such editing scopes, the recent development of novel or modified Cas-based systems has spawned an array of additional biotechnological tools, empowering both fundamental and applied research. Precisely targeting DNA or RNA sequences, the CRISPR/Cas system has been harnessed in fields as diverse as gene regulation, deepening insights into gene expression, epigenetic changes, genome spatial organization, and chromatin dynamics. Furthermore, it aids in genome imaging and sequencing, as well as effective identification and countering of viral pathogens in plants and animals. All in all, the non-editing aspect of CRISPR/Cas exhibits tremendous potential across diverse domains, including diagnostics, biotechnology, and fundamental research. This article reviews and critically evaluates the primary CRISPR/Cas-based tools developed for plants and animals, underlining their transformative impact.

## 1. Introduction

Since its inception in 2012, with the notable publication by Doudna and Charpentier [1], the **CRISPR/Cas system** has harbored the potential to catalyze an extraordinary paradigm shift in genome editing applications such as gene therapy and agricultural breeding. This technology has partly superseded transcription activator-like effector-mediated techniques (TALEN) and zinc-finger proteins. Indeed, since the **specificity of CRISPR/Cas target** is governed by **nucleic acid base pairing**, no specialized expertise in engineering DNA-binding proteins is required to be able to make use of it [2,3]. Originally, the CRISPR/Cas system evolved as a prokaryotic adaptive immune system to combat mobile genetic elements such as bacteriophages and plasmids. Archaea and bacteria exhibit a notable variation in the sequences of their respective Cas proteins and genomic loci compositions and structures. The growing knowledge regarding this diversity is acquired through screening of the constantly expanding databases of genomic and metagenomic data. The newest classification includes **two classes**, **six types**, and **33 subtypes**. Class 1 (not discussed here) includes types I, III, and IV, whereas class 2 encompasses types II, V, and VI [2,4,5].

While type VI is a recent discovery, type II has been already extensively investigated: it represents the most widely exploited group of Cas proteins and contains the Cas1, Cas2, Cas4, and Cas9 genes [5,6,7]. The Cas12 family coincides with the type V and it is further divided into 14 subtypes (Cas12a-Cas12n) [8], with Cas12a being one of the most exploited Cas proteins (together with Cas9) for genome editing approaches. Within the Cas12 family, Cas12f (also known as Cas14) is also of particular interest due to its small size (around 550 amino acids). Indeed, minimizing the length of the genes coding for the nucleases represents a fundamental step to facilitate packaging for following delivery into the cellular system. This nuclease can target both dsDNA and single-stranded DNA (ssDNA) using a single recombination UV C (RuvC) nuclease domain. Finally, worthy of note is also the discovery of Cas13a (also referred to as C2c2) belonging to type VI and isolated in *Leptotrichia shahii*, which is able to recognize and cleave single-stranded RNA molecules. Investigations into Cas14 and Cas13a could be economically valuable in engineering interference against plant ssDNA or RNA viruses.

The genome editing applications of CRISPR/Cas technology, such as gene therapy and agricultural breeding, have sparked significant investments and strategic partnerships across various industries including pharmaceuticals, agriculture, food, and biotechnology. Nonetheless, a great number of non-editing applications related to the use of modified CRISPR/Cas systems have been raised in the last few years. As deeply elucidated in the sections below, most of these non-editing systems rely on Cas enzymes that are deprived of their catalytic activity domain and therefore commonly referred to as “dead” (i.e., dCas). Among these, the corresponding “dead” versions of Cas9 and Cas13 enzymes have been exploited extensively in numerous research approaches.

While the potential for genome editing using CRISPR/Cas systems cannot be understated, it is imperative to promote a more balanced exploration of **non-editing applications**. In this review, most of the recent non-editing—here understood as those not modifying the nucleotide sequence—CRISPR/Cas-based tools developed in plant and animal research will be reviewed and critically discussed. Moreover, considering that most of the non-editing applications of CRISPR/Cas systems have been developed in animal research, we would like to focus on the technological transferability of these tools to other kingdoms, particularly in plants. After a detailed description of a set of approaches, a final consideration on this topic will be drawn.

## 2. Overview of the Main CRISPR/Cas Systems That Are Exploitable for Both Editing and Non-Editing Uses

Although non-editing applications of the CRISPR/Cas system encompass a diverse array of Cas enzymes, they mostly gravitate towards a restricted group of enzymes. Notable among these are Cas9, Cas12, and Cas13, including their non-catalytically active variants like dCas9 and dCas13. To streamline comprehension and due to spatial limitations, a concise description of these enzymes follows, highlighting specific attributes rendering them particularly adept for non-editing purposes. These attributes encompass factors such as protein size, the nature and quantity of nuclease sites, and associated mutations that render the enzyme incapable of cleaving DNA/RNA. Additionally, their interaction with precise RNA guides and the requisite single-guide RNA (sgRNA) components for targeting activities are crucial elements underpinning their utility in non-editing applications (Table 1).

### 2.1. CRISPRCas9 and dCas9

**Figure 1:**
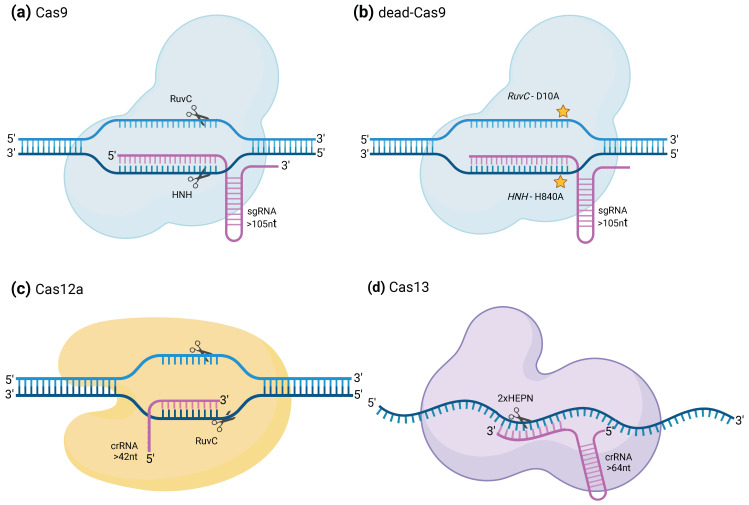
Overview of the main Cas proteins reviewed in this article. (**a**) **Catalytically active Cas9-sgRNA ribonucleoproteic complex** and how it binds a DNA target, one for each DNA strand. The length of the sgRNA is indicated. The scissors represent the cleaving sites of the RuvC and the HNH nuclease domains. (**b**) **“dead” Cas9-sgRNA ribonucleoproteic complex**. The stars represent the two mutations that induce loss of nuclease activity. (**c**) **Catalytically active Cas12a ribonucleoproteic** complex and how it binds to the target locus. The length of the sgRNA is indicated. The scissors represent the two cleaving sites of the two RuvC nuclease domains. (**d**) **Catalytically active Cas13 ribonucleoproteic complex** and how it binds to the single-stranded RNA (ssRNA). The scissors represent the cleavage site of the HEPN nuclease domain.

**CRISPR/Cas9** is probably the most well-known CRISPR system. It relies on the activity of **Cas9**, a class 2 type II Cas protein (Figure 1a). These Cas proteins require the presence of a **protospacer adjacent motif** (hereafter referred to as **PAM sequence**), close to the target sequence to recognize it and contain the **RuvC** and **HNH** (histidine–asparagine–histidine) endonuclease domain **nuclease domains**, which are involved in the cleavage of the target DNA [1,9]. SpCas9 from *Streptococcus pyogenes*, which was the first Cas9 to be used in non-prokaryotic cells and still the most widely used one, needs to recognize a NGG PAM motif to bind and cleave the target sequence. The need for a PAM sequence can be considered an obstacle to the use of the CRISPR/Cas9 system to target every possible sequence and scientists have therefore already generated **variants** from the Cas9 that recognize another or several other PAM sequences. For example, GAT, GAA, and even a binucleotidic NG can serve as a PAM sequence for the xCas9 variant. It is also possible to search living organisms for orthologues of the SpCas9 [15]. Indeed, in various species, the pattern identified as a PAM sequence for Cas9 recognition may vary and some of them may be more adapted to specific target sequences. Additionally, in certain instances, the PAM can exhibit a certain flexibility, allowing multiple variations for a particular position. For example, the PAM sequence recognized by the Cas9 orthologue of *Campylobacter jejuni* is NNNVRYM, where Y can be either C or T; V can be A, C, or G; and M can be A or C. This CjCas9 is also one of the smallest Cas9 orthologues, being only 984 amino acids in length compared to the 1368 residues of the SpCas9 [16]. Reducing the size of the Cas9 is indeed a way to facilitate their delivery into the target cell [2].

The target sequence is specified by the **guide RNA** (**gRNA**) or **single guide RNA (sgRNA)**. In the case of the Cas9 protein, the gRNA is composed of two distinct parts, often fused together in a long gRNA when engineered in laboratories. The first part is the **CRISPR-RNA** (**crRNA**) that allows **targeting of the DNA** portion corresponding to its complementary sequence. The other part is the **trans-activating CRISPR RNA** (**tracrRNA**), which allows the formation of the **ribonucleoprotein (RNP) complex**. In *Streptococcus pyogenes*, sequences encoding diverse crRNA can be chained at the same locus of the genome, in the form of an **array of crRNA spacers** separated by identical direct repeats. The transcribed long pre-crRNA can then be processed into the different mature crRNA by the SpRNase III. Each crRNA may then be assembled to the Cas9, provided that it is already binding to the tracrRNA part of the gRNA [3]. In the case of sequence targeting, whether for a genome editing purpose or not, this could allow for the introduction of different gRNAs in a unique construct and send the Cas9 to several loci at the same time. Versions of the Cas9 protein that are **deprived of their catalytic activity** (dCas9) have been developed to be able to target DNA sequences using a specific guide without cleaving them. Generation of such proteins was conducted by inserting two point mutations in its DNA sequence to introduce two substitutions in the protein chain, D10A and H840A, that induce loss-of-function of the RuvC1 and HNH nuclease domains [17] (Figure 1b).

### 2.2. CRISPR–Cas12a

**Cas12a**, also referred to as Cpf1, differs from Cas9 in the sense that it cleaves DNA forming **5′ overhangs** and not blunt ends, which can be more convenient for certain applications of genome editing like the insertion of a DNA sequence at a precise position (Figure 1c). As Cas9, it requires the presence of a PAM sequence upstream of the target sequence. As performed for Cas9, orthologues of Cas12a have been engineered to make them specific to other PAM sequences. However, and contrary to Cas9 which requires the presence of both tracrRNA and crRNA to cleave DNA, **Cas12a only requires the presence of a crRNA**, which it can process on its own from an **array of crRNA**. This reduces the length of the required oligo to order when designing the gRNA. Downstream of the *FnCpf1* gene, the locus encoding the Cas12a/Cpf1 in *Francisella novicida* U112 contains an array of several nuclease guide sequences (spacers) transcribed together as pre-mature crRNAs and interspaced by direct repeats. Cas12a is then capable of processing each one of the crRNA with **no other effector** required [2,18] while Cas9 would require the presence of the tracrRNA as well [3]. Interestingly, Cas12a not only possesses a sequence-specific double-stranded DNA cleavage activity but also a **sequence-independent single-stranded DNA degradation** property, provided that it is activated by the binding with high specificity of the DNA sequence specified by the gRNA [2,19], although only a few examples of non-editing usages of the CRISPR/Cas12a system are present in the current literature. In this review, we nevertheless tried to showcase some of the works that made use of it in order to **regulate gene expression** and **induce specific cleavage of viral DNA** in infected plant cells.

### 2.3. CRISPR/Cas13 and dCas13

**CRISPR/Cas13** systems from class VI are distinguishable from the other CRISPR systems in the sense that they naturally recognize and cleave **ssRNA** molecules according to a gRNA (Figure 1d). Contrary to DNA targeting Cas proteins, Cas13 systems do not need to recognize a PAM sequence in order to cleave its target RNA [12]. This characteristic was seen in different RNAs targeting CRISPR/Cas systems [20]. Similarly to Cas12a, activation of Cas13 proteins through recognition of the target sequence induces their “**collateral cleavage**” activity, which allows them to also cleave nearby ssRNA specifically at uracil bases regardless of the rest of the sequence. This property has been employed to develop a very quick and specific virus detection technique called specific high-sensitivity enzymatic reporter unlocking (SHERLOCK) [2,14]. Different Cas13 subtypes exist, namely Cas13a, Cas13b, Cas13c, and Cas13d, that have been established according to phylogenetic analysis of their effector complex [21]. Furthermore, for the same subtype, different orthologues from different species have often been compared for the same experiment to find the most suited one [21,22]. Catalytically inactive (“dead”) versions of Cas13 systems, especially Cas13a orthologues, have also been engineered by replacing the arginines in positions 474 and 1046 of the two **HEPN** domains with alanines [13]. Here, we show how the CRISPR/Cas13 systems have been utilized over the last decade to **label RNA**, **modify mRNA translation** in mammals, **detect the presence of targeted RNA** in samples, or even **induce resistance against viruses** in plants.

## 3. CRISPR/Cas-Mediated Transcriptional Regulation

**Figure 2:**
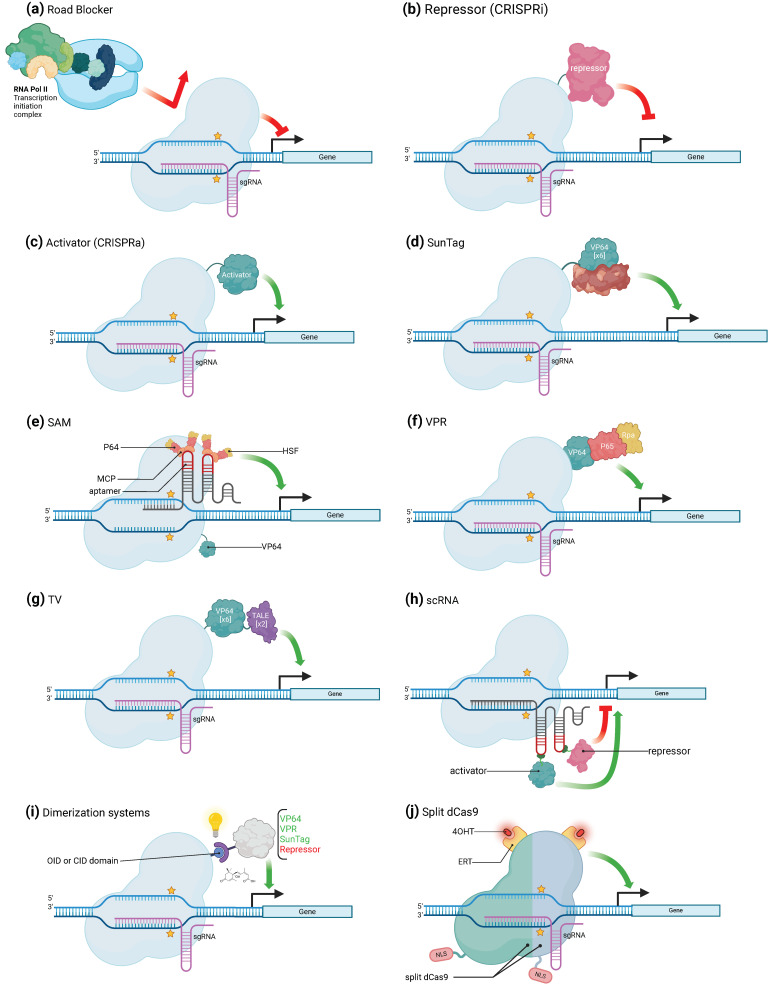
CRISPR/dCas9-based systems for gene expression regulation. (**a**) **Road blocker**: dCas9 exerts steric hindrance towards RNA polymerase by binding target DNA. (**b**,**c**) **Generic activator** or **repressor** (CRISPRa or CRISPRi): dCas9 protein is associated with a single activator or repressor. (**d**) **SunTag**: tandem array of peptides engages several copies of VP64 activator. (**e**) **SAM** (SAM: Synergistic Activation Mediator): two MS2 RNA hairpins each carrying p65 and HSF1 activators. (**f**) **VPR**: VP64, p65, and Rta activators are placed in a specific order to maximize gene activation. (**g**) **TV**: six copies of TALE and two of VP64 activators. (**h**) **scRNA** (scRNA: scaffold RNA): any combination of activators and repressors enabled by coupling several RNA hairpins. (**i**) **Dimerization Systems**: dCas9 activity spatially and temporally controlled by sensing optical and/or chemical inputs. (**j**) **Split-dCas9**: The dCas9 is divided into two separate parts that are fused to an estrogen receptor (ERT). Upon the reception of 4-hydroxytamoxifen (4OHT), the two parts are sent to the nucleus thanks to their nuclear localization system (NLS) and assemble with each other and the sgRNA to reconstitute the full ribonucleoprotein that can bind to DNA. In all panels, stars correspond to the point mutations inactivating the RuvC domains of Cas9.

In eukaryotic cells, an important part of gene regulation occurs through **transcription factors**, which are proteins that bind to specific DNA sequences, often called **cis-acting regulatory elements**, and activate or repress the expression of genes under the control of these sequences [23,24]. Over the past decade, a couple of **artificial gene regulators based on CRISPR/Cas systems** have been generated, taking advantage of the ability of Cas proteins to bind nucleic acids in a **sequence-specific** manner. Such systems have already been used to induce either **transcriptional activation or repression** of specific genes (Figure 2a–j). When used to activate transcription, the complex binds to a specific promoter region and recruits transcriptional activators to enhance gene transcription. This approach involves fusing a transcription-activating domain to the Cas protein, which allows it to recruit transcriptional machinery and activate gene expression through a specific and purposely designed sgRNA. In the same way, a transcriptional repressor domain can be fused to the Cas protein. When such a CRISPR/Cas complex is sent to a target DNA sequence using a specific gRNA, it binds to the promoter region and recruits transcriptional repressors, which represses gene expression. Both these approaches give access to precise control over gene expression through the direct target of specific DNA regulatory sequences. In this context, it is often more convenient to use dCas proteins that are **deprived of their cleavage activity**, instead of regular Cas, so that they are not molecular scissors anymore but rather **generic RNA-guided DNA-binding proteins**. To date, CRISPR systems based on Cas9 are the most employed ones in the available scientific literature. Therefore, the majority of tools to modulate gene activation and repression that will be reviewed here make use of the dCas9 protein. CRISPR systems based on other Cas proteins (namely Cas13, Cas12a, or their “dead” versions), were nevertheless used for this purpose but in a more exceptional way. This is why we chose to review them in a separate way (Section 3.3).

### 3.1. CRISPR/Cas Mediated Gene Activation

Fusing a dCas9 protein to transcription regulation effectors results in the generation of an **artificial transcription factor** able to pair with a specific sgRNA that can be **programmed for a specific purpose**. Bikard and colleagues demonstrated the potential of this molecular strategy by describing (or reporting) an example of this in which they combined the ω subunit of RNA polymerase (RNAP) with a dCas9, which resulted in the up to three-fold over-activation of a reporter gene in *Escherichia coli* [25]. Arises in this way the true possibility to use the CRISPR system for gene activation (**CRISPR activation**, or **CRISPRa**). In eukaryotic cells, one of the first successful artificial upregulation complex to be developed is referred to as **dCas9-VP64** (Figure 2c). It is a fusion between the dCas9 protein and the VP64 activator, which is a synthetic homo-tetramer of four *Herpes simplex* VP16 transcriptional activator domains from the *Herpes simplex virus*. Another early-developed fusion complex is the **dCas9-p65**, where p65 is also a transcription-activating domain. The dCas9-VP64 fusion has, however, been more frequently and more successfully employed than its p65 fusion equivalent. Several studies have shown that dCas9-VP64 can either upregulate previously activated genes or activate silent endogenous reporters [26]. Cheng and colleagues used several sgRNAs tiled all over the promoter at the same time and obtained an over-activation of the reporter gene of up to 10 folds, proving that the expression of the target gene may depend on the **copy number** of activators [11]. In addition, the position of the binding sites regarding the TSS of the gene is also crucial since the highest activation was obtained using several sgRNA localized within 300 bp upstream of the TSS [11]. Another strategy to increase the level of activation is to directly act on the copy number of an activator domain that the dCas9 carries. For example, Tanenbaum and colleagues developed the **dCas9-SunTag Platform** (Figure 2d), where the SunTag is a repetition of GCN4 proteins that are attached to the dCas9 and recruits several VP64 activator proteins that bind to it through an anti-GCN4 antibody peptide [27,28]. The dCas9-VP64 effector induced a 2-fold increase in target gene expression; using SunTag allowed the enhancement of the production of the targeted gene’s product by 50-fold [26,29]. Moreover, the dCas9-SunTag system was adapted in *Arabidopsis* for targeted gene activation [30]. Furthermore, a modified and more efficient version of the dCas9-SunTag Platform was developed, in which the SunTag not only recruits VP64 effectors but also **TET1** effectors [27,31]. With both of them, the upregulation of several genes was 212-fold on average, while it was only 20-fold with only TET1 and even less with only VP64 [27,31]. A similar synergy between effectors was also seen using a **tripartite** “**VPR**” activator composed of VP64, p65, and Rta (another activator) linked in tandem (Figure 2f). dCas9-VPR was indeed able to amplify the activation of endogenous genes by up to 300-fold compared to dCas9-VP64 alone [32]. The dCas9-VPR system was also seen to induce high transcriptional activation in tobacco (*Nicotiana benthamiana*) [33]. A similar strategy was followed to develop a new approach called “**Synergistic Activation Mediator**” (**SAM**) which, like the previous system, combines several transcriptional activators to increase the expression level of the targeted gene (Figure 2e). The base of this complex is a dCas9-VP64 associated with a sgRNA, itself containing two copies of an **MS2 phage RNA hairpin aptamer**. Each one of these hairpins is bound by an **RNA-binding MS2 coat protein MCP**. In turn, the MCP protein is fused to the p65 activator, which is linked to the activation domain of the human heat shock factor 1 (HSF1). Each hairpin motif of the sgRNA is able to be bound to up to two of such activation modules, for a total of four modules per dCas9-VP64 [34]. Finally, dCas9-TV, a new CRISPRa system, was specifically developed for plant species (Figure 2g). It relies on **TV**, an activator composed of six copies of the **TALE transcription activation domain** (**TAD**) motif and two copies of the VP64 activator. Application of this system in *Arabidopsis thaliana* and rice induced a strong transcriptional activation compared to the canonical dCas9-VP64 system [35].

### 3.2. CRISPR/Cas-Mediated Gene Repression

The first attempts to use the dCas9 for gene repression were not based on the fusion of transcriptional repressors to the dCas9 protein but simply on the **steric hamper that the protein exerts on the RNAP** when bound to the target DNA (Figure 2a). This system, referred to as **CRISPR interference** (**CRISPRi**) [17], was described to be effective in bacteria. In *Escherichia coli*, CRISPRi displayed an up to 300-fold highly specific and revertible gene repression capacity. Its efficiency, however, depends on the location of the targeted site: the highest efficiency was obtained when dCas9 was sent to either the -35 box of the promoter or the beginning of the coding sequence. Initiation or elongation of the transcription by RNA-polymerase II is blocked by the presence of the dCas9 and the **R-loop** formed by the sgRNA-DNA interaction. CRISPRi also offers the possibility to effectively downregulate several genes and this, with no crosstalk between the different inserted sgRNAs [17]. It could, however, only induce a mild transcription repression in eukaryotic cells. Indeed, in eukaryotes, simple steric hindrance is not sufficient to fully inhibit the activity of the RNAP. To overcome this issue, several enhancements have been developed. Indeed, in mammalian cells and as it has been conducted for transcription upregulation approaches, dCas9 was fused to domains that induce the recruitment of chromatin modifiers but in this case with transcriptional downregulation activity. The regulators used as proofs of concept included the **KRAB** (**Krüppel-associated box**) domain of **Kox1**, the **CS** (**chromoshadow**) domain of HP1α, the WPRW domain of Hes1, and four consecutive copies of the **mSin3 interaction domain** (**SID4X**) [36,37]. With this system, repression rates ranging from 90% to 99% have been observed in mammalian cells [36,37].

In plants, the application of CRISPRi is limited to a few specific instances. A transcript level reduction of about 40% was observed in *A. thaliana* and *N. benthamiana* using the two transcriptional repressors **dCas9-3xSRDX** (SUPERMAN Repression Domain X) and **dCas9-SRDX**, respectively [38,39,40] (Figure 2b).

### 3.3. Alternative Uses of CRISPR/Cas for Transcriptional Regulation

One of the other approaches to induce expression regulation again relies on the **in-situ assembly of regulatory platforms**, each made up of one dCas9 protein associated with the sgRNA that allows the recruitment of chromatin remodelers. These changes closely associated with the conformation of chromatin are commonly known as epigenetic modifications and often result at the base of gene expression alteration, which could also be the cause of inaccessibility to wild-type CRISPR systems. An additional alternative use of dCas proteins for transcriptional regulation can be the innovative combination of them to epigenetic modulators, in order to send relevant regulatory domains to specific regions of the genome without inducing DNA edition [41]. CRISPR/dCas9 is frequently used to control gene expression and cause epigenetic change; it is crucial for gene silencing, activation, and identification of gene functional elements as well as to investigate epigenomes.

The core of this system is then the guide RNA since it relies upon both the choice of the target and the type of regulation that is applied to this latter. The sgRNA harbors one or several hairpin structures, which form a **scaffold RNA** (**scRNA**) to which the chromatin remodeler can bind (Figure 2h). The assembly of several parallel dCas9-RNA platforms allows for the simultaneous regulation of more than one target gene. Indeed, sgRNA recognizing several protospacers can be inserted at the same time to target several genes. Plus, different types of RNA binding proteins, which can each detect a specific RNA hairpin configuration, are made use of, which induce the recruitment of specific types of effectors. This makes it possible to recruit specific combinations of effectors to the targeted genes to modulate their expression with precision. This approach allows then for a **completely programmable regulation** of each of the chosen genes in a **contemporaneous** and **independent** way [29].

A further refinement of that method was performed using ligand-inducible control of gene expression. In detail, the dCas9 is engineered so that its catalytic activity is only enabled upon treatment of the cell with a specific ligand or after detection of native **cellular or microenvironmental signals**, allowing for spatial and temporal control of gene function through sense input signals. To do so, two main methodologies have been developed, namely coupling dCas9 to chemical or optogenetic sensing or to ligand-sensing receptor domains. **Chemically-induced dimerizing domains** or **optogenetically inducible dimerizing domains** (respectively, **CIDs** and **OIDs**) have been linked to dCas9 and its corresponding regulator domain (activation or repression domain) (Figure 2i). CIDs and OIDs can dimerize when their ligands are present, recruiting the effector domain to the dCas9 binding site on the genome [42,43]. In this context, several CIDs and OIDs have been coupled with the dCas9-effector complex in order to obtain an induction triggered from stimuli of various kinds, e.g., abscisic acid (ABA)-inducible ABI–PYL1 [44,45,46], gibberellin (GA)-inducible GID1–GAI24 [46], rapamycin-inducible FKBP–FRB [44], blue light-inducible CRY2-CIB1, Magnet pMag–nMag, and phytochrome-based red light-inducible PhyB–PIF [47,48,49].

A ligand-inducible control of Cas9 or dCas9 is also feasible by engineering it into a **split-Cas9 or split-dCas9** version. In this case, the Cas9 is translated as two separate domains, N-Cas9 and C-Cas9. Each of these is fused to an estrogen receptor, which interacts with an Hsp90 chaperone. Both parts of the Cas9 are then sequestered into the cytoplasm and are catalytically inactive. Upon capture of the **4-hydroxytamoxifen** ligand by the ligand-binding domain **ERT** from the estrogen receptor, the interaction between the latter and Hsp90 is disrupted and the parts of the Cas9 can localize to the nucleus and reform the complete active ribonucleoprotein along with the RNA (Figure 2j) [43,50]. The **split-dCas9** has also been fused to the previously described CID and OID [43].

When it comes to suppressing expression without inducing modifications on the genomic sequence, another strategy could be to knock down genes through the **direct targeting of mRNA transcripts**. It was shown that, apart from double-stranded DNA, the Cas9 protein can recognize single-stranded RNA and cleave it. Interestingly, the presence of a PAM sequence on the ssRNA is still mandatory to activate the nuclease activity but it may be incompletely annealed with the sgRNA. Thus, introducing a mismatch in the PAM sequence allows the Cas9 to perfectly recognize the mRNA target while forbidding the cleavage of the corresponding DNA sequence [10]. More recently, the ssRNA-specific Cas13 was used in bacteria to generate a knockdown of specific genes through degradation of their transcript RNAs [20,51], with no required recognition of any PAM sequence. The only issue regarding the use of Cas13 orthologues is their **collateral sequence-unspecific** cleavage of ssRNA upon recognition of the target, which could cause cellular toxicity. The introduction of R597A and R1278A mutations in the HEPN domain was, however, seen to suppress the collateral cleavage activity [20]. It was also possible to target and cleave gene transcripts with Cas13 orthologues in eukaryotes. shRNA interference and Cas13-mediated knockdowns both yielded comparable efficiency in terms of mRNA quantity depletion. However, Cas13 was more sequence-specific and induced almost no off-target effects. Furthermore, developing this technique can allow for **gene knockdown in procaryotes** where RNA interference does not exist [20,51]. Other techniques make use of the catalytically inactive dCas13 to target RNA and recruit RNA-modifying effectors to it. This strategy echoes the one we described earlier for the modulation of gene translation. Recently, the **adenosine deaminase gene** (**ADAR2**) **catalytic domain** has been linked to dCas13b, allowing for programmed base editing of mRNA in human cells and the engineering of target gene expression without irreversibly altering the coding DNA [12]. The same Cas13 complex was also described to yield good results in yeast and zebrafish embryos [52,53]. In plants, the Pol II promoter is used to drive the expression of both Cas12a and its crRNA. The crRNA is flanked by hammerhead and hepatitis delta virus ribozyme RNAs for precise crRNA processing. With this system, a 90% gene expression decrease was achieved in *A. thaliana* [54].

Transcriptional modulation mediated by CRISPR/Cas-derived systems, in terms of both activation and inhibition, results in a transient modulation, which means that the transience cannot be observed in the post-mitotic cells. To maintain these changes as transferable, epigenetic modifications can be locally induced, through the addition of methyl groups to DNA or of acetyl or methyl groups on histone residues, which are the base of a gene expression modulation [55]. The induced gene expression perturbation can be passed down to the offspring cells because these changes are frequently long-lasting. Interesting strategies applied to induce persistent epigenetic modifications were especially exploited in therapeutic research [56], in which several CRISPR/Cas-derived complexes have been assembled to induce targeted (epi)modifications at the DNA or chromatin level [57]. CRISPRoff [58] and CRISPR-KAL [57] systems represent two model examples of CRISPR-based epigenetic regulation, able to lead to long-term gene silencing by modifying H3K9me3 and DNA methylation, respectively. In addition, to achieve long-term gene silencing by DNA methylation or to reverse the silencing effects of natural DNA methylation, several approaches have been developed by assembling dCas9 to domain derived from the DNA methyl-transferase 3 (DNMT3) [59] and the methylcytosine dioxygenase 1 (TET1) catalytic domain to selectively remove DNA methyl groups and upregulate gene expression [60,61].

Similar to other CRISPR tools, the dCas9 protein was fused to catalytic core domains able to drive the addition/deletion of the acetyl group in order to modify histones in a site-specific way, with the purpose of examining how epigenetic changes affect biological functions in animals. dCas9-Histone deacetylase has the ability to cause deacetylation of the genome, resulting in a general transcriptional inhibition, whereas the acetylation of lysine 27 of histone H3 (H3K27ac) results in activation of gene expression [62].

## 4. CRISPR/Cas-Mediated in-Depth Study of Gene Regulation

As described up until here, gene regulation can occur at transcriptional and posttranscriptional levels and both these are efficient pathways to target in order to develop CRISPR/Cas system-based gene regulation modulators [13,26,29]. The CRISPR/Cas system can not only be used to modulate gene regulation in experimental systems but also to study the endogenous one at both these regulation levels (Table 2).

### 4.1. Study of Gene Regulation at the Transcriptional Level

A few years ago, Liu and colleagues developed a **CRISPR affinity purification in situ of regulatory elements** (**CAPTURE**) technique, a CRISPR-based technique to identify the **DNA-binding proteins** and **chromatin regions** that are interacting with a given locus at a given time. It relies on the use of the **biotinylated dCas9** protein that is sent to a locus of interest, which is specified by a sgRNA. The chromatin is then crosslinked, sonicated, and pulled down using streptavidin beads to which the biotinylated Cas9 binds. At that point, the elements that were in interaction with the locus as the crosslink was induced are all fixed to the dCas9. After reverse-crosslink, it is possible to reveal the identity of the protein fraction that has been precipitated along with the dCas9 by performing proteomic analysis. It is also possible to have access to the distant chromatin portions that are in **long-range interaction** with the locus through the 3C-seq technique [63,64]. The authors first confirmed the feasibility of the technique with a human telomere-specific sgRNA and then tested it on single genes. They were able to precipitate the protein factors bound to single regulatory sequences of the human β-globin genes, with almost no detectable off-target effects. Moreover, simultaneous use of different sgRNAs is possible, which allows the study of different regulatory sequences at the same time. The proteomic study of the pulled-down proteins not only revealed already known regulators of these genes but also led to the discovery of new ones. Confirmation of the discovered interactants was performed using ChIP-seq. When it comes to chromatin interactions, CAPTURE allowed us to put in evidence most of the long-range interactions but also significant intra-chromosomal and short-range ones. Very recently, Wang and colleagues developed a similar technique on a vegetal species, namely birch (*Betula platyphylla*). They targeted the four regions of the promoter of the *BpNAC090* gene and identified 32 potentially regulating transcription factors for that gene, five of which were confirmed to specifically bind to this promoter [65].

**Table 2:** Types of CRISPR/Cas systems for gene expression regulation: sum up of the main features.

Name	Description	Organism	CRISPR/Cas System	Type of Regulation	Performance	References
VP64	Single activator (VP16 or p65)	Mammalian cells and budding yeast	dCas9	CRISPRa	Between 2- and 5-fold	[3,26]
SunTag	Tandem array of peptides, which recruits several copies of VP64	HEK293 and U2OS cells. *Arabidopsis thaliana*	dCas9	CRISPRa	Up to 50-fold	[28,30]
VPR	Tripartite peptide composed by the VP64, p65, and Rta activators placed in a specific order to maximize gene activation	HEK293T and Neuro-2A cells. *Nicotiana benthamiana*	dCas9	CRISPRa	Up to 300-fold	[32,33]
SAM	VP64 and sgRNA with two MS2 on turn fused to p65 and HSF1	HEK293FT and Neuro-2a cells	dCas9	CRISPRa	Variable	[34]
TV	Six copies of TAD motif and two copies of the VP64 activator	HEK293T cells. *Arabidopsis thaliana* and *Oryza sativa*	dCas9	CRISPRa	Variable	[35]
Road blocker	Steric hamper due to simple bound of dCas9	*E. coli* and mammalian	dCas9	CRISPRi	Depends on organism	[17]
Transcriptional repressors	KRAB, CS, WPRW, SID4X, 3xSRDX, and SRDX domains	Mammalian cells. *Arabidopsis thaliana* and *Nicotiana benthamiana*	dCas9	CRISPRi	Between 40 and 99%	[36,37,38,39,40]
scRNA	Differential regulation (both activation and repression) of a set of gene targets simultaneously	Human cells	dCas9	Both	NA	[29]
Dimerization systems	Spatial and temporal control of gene function through sense input signals and generate functional outputs	HEK293T cells, mice and *Avena sativa*	dCas9	Both	NA	[2,44,46]
Split dCas9	Fusing ligand-binding domains of nuclear receptors to split Cas9 protein fragments can provide chemical control over split Cas9 activity	HEK293T cells.	dCas9-	Both	NA	[50]

### 4.2. Study of Gene Regulation at the Post-Transcriptional Level

Posttranscriptional regulation may go through the regulation of the presence and the stability of a messenger RNA (mRNA) [66], which are dependent on the post-transcription sequence modifications they bear and the effectors they are targeted by. For example, some post-transcriptional modifications are crucial for mRNA expression and stability in eukaryotes, as well as the proteins that place them, remove them, or interact with them (called “writers”, “erasers” and “readers”, respectively) [67]. CRISPR/Cas system-based tools can now be used to target specific RNAs and study such protein interactants. Two main types of systems have been developed to obtain access to the proteins that are specifically bound to a transcript [21]. The first one is to **fuse the dCas13 protein with a biotin ligase** that will mark every protein complex interacting with the RNA with biotin. These proteins are then purified using classical techniques such as precipitation and can be analyzed. The other method is to **crosslink the cell with UV** once the dCas13 has bound its target RNA to covalently bind the proteins that are in close contact with the dCas13, then precipitate the whole complex through precipitation of the dCas13. These two techniques strongly echo what was performed with the CAPTURE technique. Overall, these techniques take advantage of the **low mismatch and off-target rate** of the dCas13 that allows targeting one RNA with **high sequence specificity**. The number of proteins caught using one of these methods can be a few hundred, which makes it a very promising approach to studying RNA processing for maturation and translation. It is, however, still difficult to precipitate proteins that are only transiently bound to the RNA or to study RNAs that are present in low amounts. The efficiency of the biotin-ligase is a limit to the techniques that make use of it and this efficiency can be influenced by the context of the experiment.

Many new genetic tools are already available to study or influence gene regulation in diverse types of prokaryotic and/or eukaryotic organisms, in a very precise and controllable manner. They all rely on the capacity of the Cas or Cas-derived proteins to bind to a target that is specified by the experimenter through the sequence of the sgRNA. In this part, we reviewed how Cas proteins have been engineered into countless bi-partite molecules, molecular complexes, or platforms in the scope of associating a functional activity to the sequence recognition capacity. This approach is also the basis of all the nucleic acid imaging techniques that have been recently developed.

## 5. CRISPR/Cas System to Image Specific Portions of Nucleic Acids in Plants and Animals

**Figure 3:**
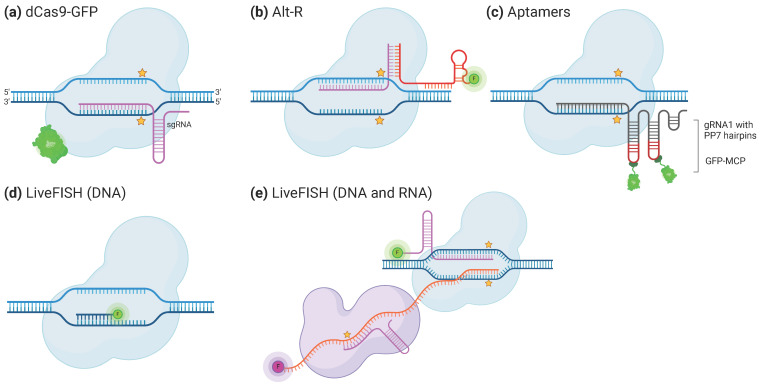
CRISPR/Cas mediated imaging of nucleic acid sequences: different tools that were developed (**a**) **dCas9 fused to GFP** and its sgRNA when bound at the target DNA sequence. (**b**) **Alt-R CRISPR/Cas9 system with dCas9**. The dCas9 is bound to DNA at the position specified by the protospacer of the crRNA part of the assembled sgRNA. The tracrRNA is bound on one side to the tail of the crRNA and to the other side is attached an ATTO fluorophore (F). (**c**) **The sgRNA is prolonged into a scaffold harboring two aptamers** to which MS2 and PP7 proteins can bind, which are themselves fused to fluorescent proteins (F). (**d**,**e**) Nascent RNA labeling **using LiveFISH**. Cas9 is guided to a transcribed DNA locus and is labeled through the fluorophore (F) carried by the sgRNA. In the meantime, Cas13 is guided to a 5′ sequence of the RNA being transcribed and is labeled through the fluorophore (F) carrying guide. The stars represent the loss-of-function mutations on the two nuclease domains.

Nucleic acid imaging is the process of visualizing and detecting nucleic acids using various imaging techniques (Figure 3). One of these techniques, fluorescence in-situ hybridization (FISH), uses fluorescent DNA or RNA probes carrying a fluorophore that binds to complementary sequences of nucleic acids, which can then be visualized under a fluorescent microscope [68]. Other methods make use of GFP (green fluorescent protein)-tagged proteins [69]. These methods use genetically engineered cells that express a fusion protein consisting of GFP and a protein that specifically binds to nucleic acids, such as a DNA- or RNA-binding protein, and allows the detection of the presence and location of the nucleic acid of interest. GFP-tagged proteins offer several advantages for nucleic acid imaging, including high specificity, sensitivity, and non-invasiveness. The ability of the Cas proteins to specifically bind to the sequence specified by the guide RNA has already been widely used to observe in-situ-stained RNA and DNA. Here, we review some of the diverse methods that have been developed to do so, may they be in fixed or living cells (Table 3). Labeling of DNA sequences using the CRISPR system has also been recently reviewed by several other groups [70,71]. The Cas proteins used for that purpose are often “dead” ones, to avoid degradation of the target sequence. It is interesting to note that instead of disabling the catalytic activity of Cas9, it is also possible to use a sgRNA whose spacer is only 11 nucleotides long, which is too short to enable the Cas-protein’s catalytic activity [72]. In most of the studies and reviews mentioned here, the targeted and stained sequences were repetitive sequences because they induce clearly visible foci of fluorescence and are therefore more suited to proofs of concepts.

One of the first approaches to stain DNA using the CRISPR/Cas system was simply to **fuse a GFP to the dCas9** (Figure 3a). This type of chimerical protein was first used about a decade ago to stain telomere sequences in live human cells [73]. Cells were transfected with an activable dCas9∷eGFP (eGFP: enhanced GFP) fusion protein along with the corresponding sgRNA leading to telomeric repeats. The efficiency of telomere detection was evaluated by counting the number of visible focal points on the imaged loci. According to the article, a modified version of the sgRNA was developed by Chen and colleagues, leading to further improvement in the precision of telomere staining and reduction of background interference. The CRISPR-staining technique then showed an **efficiency comparable to that of FISH** with a **quasi-absence of off-target**, making it a very precise way of imaging genomes. Contrary to FISH, staining with the CRISPR system does not involve cell fixation, enabling the tracking of telomeric foci movement in various situations. Chen and colleagues also applied this technique to **stain individual loci at the same time**, by inserting different sgRNA in the cell. Imaging specific genome regions can be used to study chromatin conformation at these loci but was also used to test the specificity of a novel CRISPR/Cas-based method at a glance, such as CAPTURE in human cells [63]. The dCas may also carry a **long tag**, which can serve as a binding site for one or multiple GFP-fused proteins. An example of this is the **Cas9-mediated fluorescence in situ hybridization** (**CASFISH**) technique developed by Deng and colleagues [74], which makes use of a dCas9 harboring a HaloTag. The dCas9 is then labeled using fluorophore-conjugated Halo-ligands. The whole is incubated with the sgRNA ex situ and then used to stain specific DNA sequences. Deng and colleagues tested their technique to label repetitive sequences and single loci of cells. The ribonucleoprotein complexes were also capable of penetrating in a thin mouse brain section and staining their target repetitive sequence. The protocol duration is a mere 15 min, making it a viable approach for efficient identification of the presence of a specific gene sequence in a clinical assay. Furthermore, once assembled, the sgRNA-dCas9-fluorophore complex is **highly stable,** which makes it possible to stain diverse loci at the same time, by associating each sgRNA with a different fluorophore. Pre-assembled complexes were also used to perform live imaging of specific loci through the **CRISPR live-cell fluorescent in situ hybridization** (**LiveFISH**) technique and study, among others, the recruitment of 53BP1 (p53-binding protein 1) at the double-strand break position [72]. The authors employed a sgRNA that was conjugated to a fluorophore and intentionally kept the protospacer short to prevent cutting. This allowed the use of the same Cas protein with a longer sgRNA that lacked labeling to induce a double-strand break a nearby region. Subsequently, the team was able to analyze and **quantify the recruitment of 53BP1** to the double-strand break by examining its colocalization with the initial Cas9-sgRNA complex. By employing another Cas9-sgRNA complex labeled with a second fluorophore, they could **observe a translocation** between the Chr3q29 and Chr13q34 loci and investigate the kinetics of this mechanism. The main advantage of conjugating the fluorophore with the sgRNA rather than with the Cas protein is that it allows for a **higher signal/background ratio** compared to FISH and GFP-tagged dCas labeling [72,75]. The explanation for this is that there is no signal coming from non-specifically bound dCas9 proteins. Fu and colleagues used a sgRNA harboring two types of **aptamers**, **MS2 and PP7**, in a **long 3′ scaffold** (Figure 3c). These aptamer sequences can be bound by MCP-eGFP and PCP-mCherry fusion proteins, respectively. The use of this system with the two fluorophores allowed us to **independently label two loci** of the same nucleolus [75]. The kinetics of the cellular division from the point of view of minor and major satellites in mouse fibroblast could be followed using this method. This system was also proven usable in plants and telomeric sequences of *N. benthamiana* could be stained with GFP-fused MS2 proteins binding to sgRNA and observed in live cells [76]. Though incorporating the earlier reported scaffold-modification [73] to the sgRNA resulted in a further reduction of the background signal in animal cells in mammals [75], it was not the case in plants [76]. Furthermore, in plants, the number of telomeric foci was lower than the one counted after staining with FISH, putatively because of the difference between the plant breading temperature and the Cas9 working one [76]. Such a phenomenon had previously been noticed with GFP-fused dCas9-mediated staining of telomeres [77]. Other nucleic acid imaging methods work on the staining of extracted and fixed nuclei from cell material. In **CRISPR-FISH**, also called RNA-guided endonuclease. in situ labeling (RGEN-ISL) the dCas9 is again used to target and stain specific regions of the genome thanks to a given gRNA [78,79]. An advantage of this approach is that the technique can be performed using the same protocol across a broader temperature range (from 4 °C to 37 °C) and is applicable to nuclei or fixed tissues from various species. It makes use of the **Alt-R CRISPR/Cas9** system (Figure 3b), in which the tracrRNA part of the sgRNA carries an ATT-550 fluorophore [80]. This allowed the visualization of satellite repeats and telomeric and centromeric regions of chromosomes with high fluorescence yield and low background in different plant species [78,79,81,82]. This kind of approach could be useful to study and compare the recruitment of certain proteins or the recruitment of specific histone marks at a specific locus. However, for now, the technique has not been developed for the study of single loci.

The aforementioned techniques can be categorized into two groups based on the system employed for the introduction of the Cas protein, guide RNA, and any other relevant proteins. They can first be introduced into the cell or sample as a preassembled ribonucleoprotein [72,74,78,82]. Other studies transformed their cells with one or more plasmids encoding these elements [63,73,75]. Fu et al. used a single vector encoding dCas9 and sgRNA as well as the labeling protein MCP and PCP both fused with an eGFP [75]. Likewise, the construct to express the equivalent system in plants was introduced into the plant’s genome by *Agrobacterium rhizogenes*-based hairy root transformation, the floral dip method, and via leaf samples but only transient insertion allowed correct labeling [76].

Not only DNA can be targeted by CRISPR/Cas systems but also RNA molecules. As recently reviewed by Cao and colleagues, dead versions of different Cas13 orthologues have been used to stain RNA in live cells [21]. The most common method was to conjugate the dCas13 with one or more fluorophores and to send it to a specific transcript, coding or not. For example, in mammalian-cell transformed with one vector encoding the **dead version of a Cas13** orthologue from *Leptotrichia wadei* (dLwaCas13) **fused with GFP** and a vector containing the guide RNA, it was possible to track the localization of transcripts to stress-granules, first in fixed cells but then in live cells [13]. A modified version of the **LiveFISH** technique that makes use of **both dCas9 and dCas13 with diversely labeled sgRNA** allowed DNA and RNA to be stained at the same time in live cells [72] (Figure 3d,e).

Here, we presented different techniques that, again, make use of the **highly sequence-specific binding property** of the Cas proteins to stain specific portions of nucleic acids. It also offers the possibility to image **live cells** in a lowly invasive way and with a reduced background. Such imaging of DNA or RNA is crucial to answering biological questions on important molecular processes that happen in the nucleus or quickly evidence a specific sequence. The Cas protein and its guide can be introduced into the cell through **transient expression** in a plasmid or direct transfection of the **preassembled ribonucleoprotein**. The latter is possible thanks to the high affinity of the Cas protein for its sgRNA. The advantage of directly transfecting the cells with sgRNA-Cas ribonucleoproteins is also that there is no need for stable integration of genetic constructions in the genomes.

**Table 3:** Methods making use of CRISPR/Cas systems to evidence and image nucleic acids in a sequence-specific way.

Name	Description	Organism	Type(s) of Cas Protein	Advantages	Disadvantages	Performances	References
dCas9∷eGFP fusion protein	Imaging of DNA loci with a GFP-dCas9, expressed in situ along with the gRNA from transfected vectors.	Human	dCas9	The use of an sgRNA guide with a custom scaffold reduces non-specific binding of Cas9. Possibility to label heterochromatin regions.		Labeling of repetitive sequences as well as single loci. Tracking of telomere dynamics in live cells Labeling of different positions of the same gene. Gene copy-number identification	[73]
Cas9-mediated fluorescence in situ hybridization (CASFISH)	dCas9 harbors a HaloTag flag and can be bound by fluorophores that are linked to HaloTag ligand.	Human	dCas9	Highly stable sgRNA-dCas9-fluorophore complex High specificity Several loci can be stained at the same time: multiplexed imaging. Very quick protocol (15 min) Performed at room temperature		Imaging of repetitive sequences in detection of the allele of a certain sequence in the genome of cells at a tissue scale. Dual color Genetic diagnosis	[74]
LiveFISH	One Cas9 harbors a labeled sgRNA with a short protospacer to disable cutting and one other Cas9 harbors a normal unlabeled sgRNA	Human	Cas9, dCas9, Cas13, dCas13	One type of Cas9 is used because the RNP complex is preassembled. Live imaging is possible. Combinable with other CRISPR/Cas system-based techniques.		- Used to visualize and quantify the recruitment of a protein to a specific locus and study the kinetic of such recruitment; - Used to visualize translocations; - Dual labeling of DNA and RNA.	[72]
Labeling sgRNA scaffolds in animals	sgRNA carries a long 3′ scaffold that harbors aptamers, to which fluorescently labeled proteins bind.	Mouse	dCas9	Fewer background compared to labeling with GFP-fused dCas9 because non-specific binding of dCas9 is not visible. A single vector encodes every component of the system. Live imaging is possible. Multiplex labeling	Non-specific binding is not visible, i.e., off target cannot be characterized.	Labeling of nuclear structures, repetitive sequences, and single loci. Study of chromatin dynamics during cell division. Labeling of two loci in different colors.	[75]
Labeling sgRNA scaffolds in plants	sgRNA carries a long 3′ scaffold that harbors aptamers, to which fluorescently labeled proteins bind.	Tobacco	dCas9	Fewer background compared to labeling with GFP-fused dCas9 because non-specific binding of dCas9 is not visible. A single construct encodes every component of the system. This construct is inserted with A. tumefaciens-mediated transformation. Up to 2 simultaneous labeling. - Labeling efficiency is not dependent on dCas9 gene expression level.	Non-specific binding is not visible, i.e., off target cannot be characterized. Lack of telomeric foci compared to FISH because of the working temperature in plants. Cannot be improved by modification of the RNA scaffold. Only repetitive sequences have yet been targeted. - Transient transformation of the construct is required; - Labeling efficiency is heavily dependent on the copy number of aptamers in the construct.	Live imaging of telomeric repeats in plant cells.	[76]
RGEN-ISL/CRISPR-FISH	Imaging of loci in purified fixed nuclei using a preassembled ribonucleoprotein that contains the dCas9 and its sgRNA, for which the tracrRNA part is fused to a fluorophore for labeling.	Soybean, mouse, wheat, rye, maize, and tobacco	dCas9	No plasmid construct. No in vitro RNA synthesis. Theoretically available in any species Non disruptive technique Simple and fast Usable for repetitive sequences and single loci.	Fixation of nuclei is required. ATT550-labeled tracrRNA and the crRNA that can bind to it must be ordered and are costly.	Labeling of centromeric and telomeric repeats in diverse species. Optimization of sample fixation to increase labeling yield. Time-lapse-mediated study of the binding dynamics of dCas9-sgRNA complex to DNA.	[78,79,82]
mRNA imaging	dLwaCas13 is fused to GFP expressed along with the specific sgRNA using a transient vector.	Rice, mammals	dCas13	Specific targeting of mRNA Applicable to live or fixed samples.		Imaging of a specific gene’s mRNA to track its localization at stress-granules.	[13]

## 6. CRISPR/Cas-System as a Tool to Target Viruses

### 6.1. CRISPR/Cas-System for Inhibition of Viral Infection in Plants

Before being a useful tool for scientists, CRISPR/Cas9 is, at first, a defense mechanism to counter the infection of the host cell by exogenous genetic elements, plasmids, or viruses [83]. However, viruses are not only a threat to archaea and bacteria but also to plant and animal organisms.

**Figure 4:**
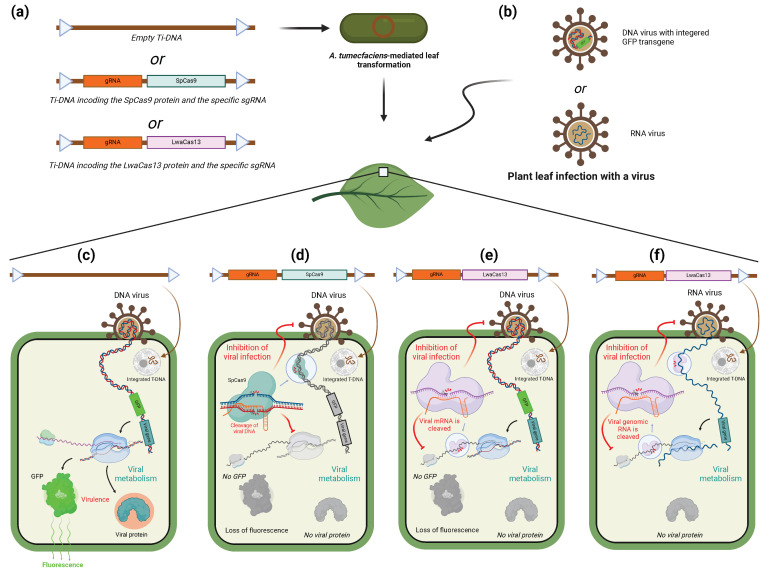
CRISPR/Cas system-mediated induction of resistance against viral infection, for example in vegetal organisms. (**a**) **A leaf is transfected** with a vector through *Agrobacterium tumefaciens*-mediated agroinfiltration. (**b**) **It is then infected with a virus.** A GFP-encoding gene can also be inserted into the viral genome. (**c**–**f**) **Experimental designs** and molecular functioning of the CRISPR/Cas-mediated resistance to virus. The vector can be either empty (**c**) or harboring a sgRNA as well as the Cas9 (**d**) or the Cas13 (**e**,**f**).

Almost a third of crop diseases could be linked to plant-infecting viruses [84]. To improve crop resistance against specific known viruses, the idea of specifically targeting viral genomes mimicking the bacterial CRISPR/Cas9 system during the infection in plants rapidly grew (Figure 4). In one of the first attempts to do so, the resistance to tobacco rattle virus (TRV) of a tobacco line expressing Cas9 at a high level was increased by previous agroinfiltration of guide RNAs specific to diverse regions of the TRV genome [85]. Amplification, cloning, and sequencing of the viral genome from such infected plants confirmed the Cas9 activity, revealing the presence of indels and point mutations in correspondence with the specific sequences targeted by the sgRNA in 42% of clones. Such examples exist for both RNA- and DNA- viruses, as reviewed by Kalinina and colleagues [86]. In the listed studies, the plant’s tolerance is improved for viruses of the Geminiviridae family using *Agrobacterium*-mediated transformation of a vector encoding a Cas protein and the gRNA targeting one or more coding or non-coding sequences of the viral genome. Whether DNA or RNA should be cleaved dictates which Cas protein should be expressed. To visualize the infection on the leaf, some studies introduced the GFP coding sequence into the viral genome. In doing so, both expressions of GFP mRNA and fluorescence on the leaf can be taken as a marker of the viral infection. In a recent study, Yu et al. used this method to study the efficiency of different types of Cas13a in conferring resistance against sweet potato chlorotic stunt virus (SPCSV) [22]. The results tend to show a greater efficiency of the LwaCas13a in reducing the expression of GFP compared to the other tested orthologues. After that, it was demonstrated that it is possible to induce resistance against TuMV in *N. benthamiana* by targeting viral RNase3 with LwaCas13a. The strategy of direct targeting of the viral genome or mRNA using a Cas13 or Cas9 echoes the use of RNA interference to induce resistance against a specific virus, reviewed by Taliansky and colleagues [87]. CRISPR/Cas inhibition is, however, limited by the fact that it requires a permanent expression of Cas and guide RNA to generate a whole resistant plant [87]. Indeed, Spencer and colleagues recently reported that the induction of RNA interference could only be observed upon stable expression of the Cas protein [88]. Compared to this, the use of RNAi to target the virus and prevent or diminish the infection only requires treatment of the plant with interferent RNA. As previously seen, targeting of RNA by the CRISPR/Cas system is very specific and less subject to off-target effects than RNAi [13]. Sometimes, as in the case of an attempt at inhibiting SPCS virus, CRISPR/Cas was the only of the two methods that was even able to give satisfying results [22]. While the high specificity of CRISPR/Cas systems may be seen as an advantage of CRISPR/Cas mediated inhibition, it makes it extremely vulnerable to mutations of the virus it is supposed to give the plant resistance from. Though out of the scope of this review, it is to be said that the editing function of the CRISPR-Cas can be employed to target and induce mutation on genes of the host plant that confer a susceptibility to infection by viruses or other pathogens [89,90].

### 6.2. Use of CRISPR/Cas System to Target Viruses in Animals

Inhibition of viral infection with CRISPR-Cas systems has also been explored in animal and human medicine. Numerous studies have already demonstrated the downregulation of viral infection in animal cell cultures. These studies have employed various types of Cas proteins depending on whether the targeted virus is based on DNA or RNA [91,92]. For example, CRISPR/Cas9 and Cas12a were shown to downregulate the infection of Green Monkey kidney cell culture by Herpes Simplex Virus 1 (HSV-1) [91]. In this study, the authors targeted the HSV-1 genome with a Cas9 or Cas12a protein and a gRNA specific to a viral DNA polymerase and were able to see a clear diminution of the infection of the cell culture as well as an immunity that lasted for several days. The efficiency of the inhibition and the duration of the conferred immunity, however, highly depended on the protospacer that is targeted by the Cas protein. Plus, the efficiency of targeting also depends on the type of Cas used, which shows that independently of their differences in PAM sequence, the Cas orthologues have diverse requirements in terms of chromatin context. On top of that, the sites targeted by Cas12a seem to display a lower mutation rate, which makes it more difficult for the virus to develop a resistance against Cas12a than against Cas9. However, Cas9 exhibited an extended duration of immunity in the observed cases. As in plants, it is possible to use other types of Cas proteins, namely Cas13, to target RNA viruses. The use of Cas13 to target viral genomic RNA or mRNA has already been extensively reviewed [92]. In this review, the authors describe different studies that were in animal cell cultures infected by a virus, majorly with SARS-CoV-2 and influenza, whose genomic RNA and mRNAs were targeted by different Cas13 proteins. LwaCas13a, LbuCas13a, PspCas13b, and Cas13d were all found to downregulate viral infection and in some cases conferred resistance for several days. Concerning their efficacy, Cas13b seems to be as efficient as shRNA interference, while Cas13a could induce superior efficiency. Concerning the therapeutic usage of the CRISPR-Cas system in animals, the way to administer the RNP and the immune reaction it may induce come into question. Viral transfection is the most used method to deliver the protein but it is also important to consider the fact that the viral vector itself can induce an unwanted immune response in the body or have a cytotoxic effect in the case of cell cultures. The 13d orthologue of the Cas13 could be used because of its smaller size and therefore should be prioritized. Transfection of RNP has the advantage of preventing the introduction of exogenous genetic sequences in the host’s cells but it could be also possible to use synthetic RNAs encoding the protein and its gRNA. Still, one remaining main issue is the potential immune response induced by the RNP itself. Strategies to minimize this mainly rely on masking the bacterial antigens that are found on the protein, by either removing the antigens that can be removed without disturbing the enzyme’s activity or by adding some antigens that downregulate the adaptive immune response. Another point that is mentioned in the article is the usage of Cas13 to diagnose viral infections. The SHERLOCK system uses the property of the Cas13 that makes it able to cut RNA randomly once it has bound its target RNA specified by the gRNA. The sample undergoes a T7 transcription to amplify the eventual RNA virus and then the Cas13 is added with a gRNA specific to the targeted virus, as well as an RNA-bearing fluorophore. If the target virus is bound by the Cas13, it will cut the fluorophore-RNAs. Then, a kind of ELISA test is performed to separate and reveal the cut fluorophore-RNAs. The SHERLOCK technique has been further developed and its variants use different orthologues of the Cas13 to allow for simultaneous analysis of different viruses. dCas13 can also be fused to other proteins to mark viral RNA (GFP), modify its epitranscriptome (mainly m^6^A), base-editing, and modulation of viral RNA splicing. This can be used to inhibit viral infection or to better understand the host–pathogen interaction.

CRISPR/Cas-mediated resistance to viruses is a growing trend that is using different approaches to target and counter this type of pathogen. Its development requires consideration of the variety of viruses and the variety of hosts they can infect. In plants, the method is still in its embryonic phase and needs further improvement before it can be applied to crops, independently of legal restrictions. Though it still faces disadvantages compared to RNAi inhibition, it remains a promising approach. Research for the use of CRISPR-Cas systems against animal viruses, including human ones, probably benefited recently from the high need for knowledge on human viruses due to the global pandemic and is therefore a little more advanced than it is for plants. Different aspects of the fight against virus infections have already been looked at, from the targeting of the genomic nucleic acid to the one of acid messenger RNA, to the detection of viral traces in samples. It will hopefully not take long before it is possible to do so in plants as well.

When it comes to the detection of nucleic acids from non-viral pathogens, the panel of available and confirmed techniques is much reduced but some authors did report some of them [93,94]. They often base themselves on the unspecific cleavage of a DNA fragment carrying one or two fluorophores, triggering the emission of light from them. For example, a modified version of SHERLOCK was used to detect *Plasmodium* parasites. Introducing PCR amplification cycles in the process could also increase the sensibility of the tool and lead to the detection of *Leishmania viannia* through its 18S ribosomal DNA gene, as reviewed in [93], or the detection of *Staphylococcus aureus* in food [94,95]. Using different probes with different fluorescent dyes, the CARMEN (CARMEN: Combinatorial Arrayed Reactions for Multiplexed Evaluation of Nucleic acids) technique and its derivates allow us to perform a multiplexed analysis and rapidly test an important number of samples for several pathogens at the same time [93].

## 7. CRISPR/Cas as an Enrichment Tool for Next-Generation Sequencing

**Figure 5:**
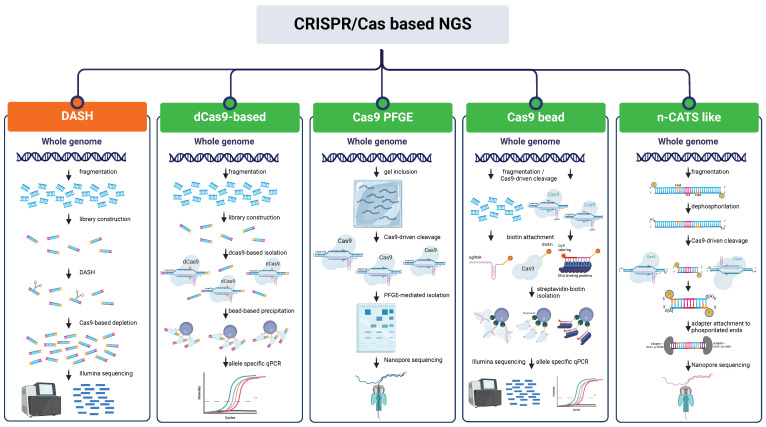
Main application categories of the CRISPR/Cas system for sequencing. Methods used to deplete the samples from unwanted sequences (orange) and enrich them in desired ones by **dCas9** or **PFGE** or bead-based isolation and **nCATS** (green) are shown.

As sequencing costs continue to decline, it is becoming increasingly difficult to avoid generating unnecessary or possibly unusable data in an experiment and, although sequencing data are easily producible in most laboratories today, the scientific community still struggles with sequence analysis. Therefore, the need to target specific genetic loci is urgent and a broadly applicable technique to screen out the signal from high abundance undesirable species before sequencing is needed [96]. Being an important support tool for sequencing, nucleic acid enrichment techniques have progressively evolved leading to the existence of a collection of methodologies applicable according to the different experimental needs (Figure 5). However, these techniques usually rely on PCR amplification to provide highly specific sequencing and are prone to allelic bias and produce non-native DNA entailing the loss of epigenetic information [97]. In the last few years, alternative tools have been developed, often in combination with the use of Nanopore sequencing, with the aim of expanding the possibilities to investigate nucleic acid features such as the observation of structural variants (SVs) at the haplotype level through long-read sequencing and detection of DNA methylation (Table 4) [98,99]. These tools exploit the CRISPR/Cas system and its two main characteristics: the high specificity of targeting through sgRNA, which also avoids long hybridization times, and the existence of a plethora of Cas proteins with the ability to perform a series of molecular actions on the nucleotide sequence of interest.

### 7.1. CRISPR/Cas NGS Approaches Based on Depletion of Undesired Region

The initial approaches to the application of the CRISPR/Cas technology started in 2016 in the realm of human diagnostics and focused more on lowering background noise while preserving the representational integrity of untargeted sequences than on the enrichment of the ROIs (regions of interest) [100]. In fact, despite the modest enrichment provided compared to other strategies, removing unnecessary sequences has proven to be one of the fastest and easiest workflows. The **DASH** protocol (**Depletion of Abundant Sequences by Hybridization**) involves attaching adaptors to DNA fragments and exploiting the specificity of sgRNA to cleave specific DNA sequences, then sequencing the remaining intact fragments using primers compatible with the adaptors [101]. On the other hand, **CUT-PCR** (**CRISPR-mediated, Ultrasensitive detection of Target DNA by PCR**) targets and cleaves wild-type DNA sequences with an intact PAM site; mutant target regions are then enriched by PCR and the process is repeated to maximize cleavage specificity prior to sequencing [102]. These technologies widely used in medical research (pros and cons are superbly reviewed by Schultzhaus and colleagues [96]) have recently been applied to plant science and metagenomics. The **Cas-16S-seq** method allows the sequencing of the hypervariable regions of the 16S rRNA gene of the microbiota by eliminating contamination due to mitochondria and plastid 16S of the host plant [103]. The variable regions of 16S rRNA are amplified in a first PCR using universal primers with adaptors, then a specific gRNA targeting the host plant’s 16S is employed for Cas9 cleavage. After cleavage, the plant’s (rice) 16S rRNA fragments are not amplified in the second PCR and are then excluded from the sequencing. In order to eliminate repetitive sequences from a genome that is particularly rich in repetitive elements, such as that of lentils, the target specificity of the CRISPR/Cas system combined with a set of over 500,000 gRNAs was used and allowed to exclude 40% of the reads mapping on repeats from sequencing and to enhance the number of reads mapping on unique regions by more than twice as much [104]. Using such methodologies allows us to focus on relevant genomic regions and to increase genotyping accuracy.

### 7.2. CRISPR/Cas NGS Approaches Based on Enrichment of Regions of Interest (ROI)

Similarly to depletion-based NGS techniques, the first advances in using CRISPR/Cas technology to selectively enrich regions of interest for sequencing purposes emerged predominantly in the field of diagnostics. High-sensitivity procedures were developed taking advantage of dCas9 and its methods of isolation. In the protocol by Aalipour and colleagues [105], the allele frequency of a rare genomic alteration causing cancer is increased through a dCas9-based method. dCas9-associated sgRNAs are designed to target mutations of interest. After incubation with DNA, the target-bound dCas9 is isolated through immunomagnetic precipitation, target DNA is then purified and analyzed through allele-specific qPCR resulting in a 21-fold enrichment. **CATE-seq** (**CRISPR-assisted targeted enrichment-sequencing**) [106] is a highly sensitive alternative approach able to reach over a 3000-fold enrichment of the target sequences. Such protocol consists of the fragmentation and subsequent specific adaptor ligation to sample DNA, targets are then bound by dCas9 and purified for allele-specific PCR or library preparation.

A few strategies are halfway between the bead-based purification of the fragments and the exploitation of the cleavage capacity of Cas9. In the ultrasensitive tool **CRISDA** (**CRISPR–Cas9-triggered nicking endonuclease mediated Strand Displacement Amplification**) [107], Cas9 cleavage activity is combined with highly specific amplification of the target site and annealing of biotin and Cy5-labeled PNA (peptide nucleic acid) probes to the amplicon to achieve attomolar sensitivity. The **CRISPR-Capr** approach [108] is based on the enrichment of cleaved target sequences using biotinylated sgRNAs and allows a 183-fold enrichment of a 13 kb DNA region. Another strategy includes DNA fragmentation by Cas9 and biotinylated adaptors are then used to repair DNA lesions [109]. At the end of the process, target sequences are long enough to be processed with long-read sequencing (20–30 kb). Finally, Tsai and colleagues [110] performed Cas9-mediated cleavage of a long sequence-PacBio-SMRT (Single Molecule—Real Time) DNA library. Bead-based isolation through adaptors led to a 64,000-fold enrichment of under-represented sequences.

In this context, the augmentation of genomic fragment lengths for sequencing purposes has become a crucial objective in genomic and functional research. Over the past few years, various approaches utilizing the CRISPR/Cas system have been devised and evaluated for TGSeq (third generation sequencing). In the case of **CISMR** (**CRISPR-mediated isolation of specific megabase-sized regions of the genome**), the isolation of the ROI through Cas9-driven cleavage at the flanking sites is combined with a pulse-gel electrophoresis step for sequence isolation and, passing through an amplification step, long-read sequencing [111]. Despite its laboriousness and time cost, this strategy proved efficient for 10-fold enrichment of sequences up to 2.3 Mb. With a similar protocol, Li et al. were able to construct the haplotype-resolved assembly of a target region through the PacBio HiFi sequencing of the CRISPR-enriched 4.5 Mb sequence [112]. Such strategies were followed by the more specific Oxford Nanopore sequencing-aimed **CATCH** (**Cas9-assisted targeting of chromosome segments**) technique, which allowed 237-fold targeted enrichment of the human *BRCA1* gene through isolation of a 200 kb region [113].

In the wake of TGSeq-aimed enrichment strategies, the finding that Nanopore sequencing adapters preferentially link to Cas9-cleaved DNA rather than artificially dephosphorylated DNA ends enabled the development of a Cas9-based enrichment approach called **nCATS** (**nanopore Cas9 Targeted-Sequencing**) was achieved [114]. Before Cas9 cleavage, pre-existing DNA ends are dephosphorylated. Next, preferential ligation of the newly produced DNA ends with adaptors is performed in preparation for Nanopore sequencing. This method yields fragments that are roughly 20 kb long, which is substantially less than the two previously stated methods but still enables 300-fold target enrichment. However, the absence of an amplification step strongly reduces process times and ensures that analyses of DNA methylation states are possible. Some protocol changes were made for the detection of genomic duplications [115], the observation of gene fusion events [116], and the reduction of background reads [117]. Exploiting the possibility of obtaining native DNA, an nCATS-like approach was used to identify the causal red fruit color variant in *Malus domestica* (Type 1 red flesh), previously defined as an 8-kb repeated mini-satellite motif upstream of the *MYB10* transcription factor. The same strategy with some implementations allowed the identification of SNPs and SVs in the *MYB10* sequence of *Prunus salicina*. Moreover, Kirov et al. were able to identify SNP and InDel variations of full-length glutenin genes using the nCATS approach *in planta*, providing helpful knowledge for marker design and leading to a definition of polymorphism at the single allele level [118]. Similar approaches are the ones based on “Negative Enrichment” [97,119] and the **FLASH** (**Finding Low Abundance Sequences by Hybridization**) strategy [120]. Taking advantage of the recently exploited Cas9-mediated adaptor ligation approach, McDonald et al. [121] combined it with a computational pipeline for the discovery of MEIs (mobile element insertions) in repetitive genomic regions in humans. In plants, Merkulov et al. [122] developed the novel **NanoCasTE** pipeline to scout both genetically inherited and somatic transposable element insertions (TEIs). The EVADÉ (EVD) retrotransposon insertions were identified in *Arabidopsis* with a 40x sequence coverage and in an *Arabidopsis* mutant with only 0.2× coverage, which is much lower than the one needed for TEI identification based on WGS (whole genome sequencing). As an additional benefit of the Cas9-mediated sequencing approaches reported here, sequencing and mapping long reads on the genome could facilitate the identification of mobile elements in highly repetitive regions like centromeres and heterochromatin. As a slight deviation from the approaches outlined above, deep sequencing of target loci is the aim of the **CRISPR-DS** (**CRISPR-Duplex Sequencing**) approach [123], which can reach up to 49,000-fold target enrichment by cleaving target DNA into short fragments. A focus on the sequencing of shorter fragments is **Cas9-tiling** [124,125]. In this case, the ROI is divided into smaller overlapping sub-regions (sub-ROI) of lengths of up to 25 kb and are enriched and sequenced separately. Finaly, in a study proposed by Lopatriello et al., the Cas9-tiling approach allowed the de novo assembly and identification of SVs of a 250-kb region on chromosome Pv05 of *Phaseolus vulgaris* [126].

**Table 4:** Cas-driven approaches aimed at sequence enrichment or depletion for sequencing.

Name	Organism	CRISPR/Cas System	Description	Pros	Cons	Desired Sequences Fold Enrichment/ Undesired Sequences Fold Depletion	Target Sequence Length	References
DASH (Depletion of Abundant Sequences by Hybridization)	Human	Cas9	DNA is fragmented, adapter ligated, sgRNA-driven cleavage, and sequencing of adapters-ligated fragments.	Highly efficient for background noise reduction	Background sequences must be repetitive; high amount of sgRNAs to be provided; amplification step needed	3–10-fold enrichment/30–105-fold depletion	2.5 kb	[101]
Cas-16S-seq	Rice	Cas9	16S rRNA amplification and adapter ligation, host’s 16S sgRNA-driven cleavage, second amplification of remaining microbial 16S, and sequencing.			8.6–22-fold depletion		[103]
	Lens culinaris	Cas9	Custom gRNAs-driven cleavage of DNA repetitive regions, PCR amplification, purification, and sequencing.			2.6-fold enrichment	2.9 Gb of repeats	[104]
CUT-PCR (CRISPR-mediated, Ultrasensitive detection of Target DNA by PCR)	Human	Cas9	sgRNA-driven cleavage of WT DNA with intact PAM site, PCR enrichment of mutants, cleavage step repeated, and sequencing.		Detected mutations must be in the PAM site; several amplification steps needed	30–600-fold enrichment	1 kb	[102]
CRISDA (CRISPR–Cas9-triggered nicking endonuclease mediated Strand Displacement Amplification)	Human	Cas9	Two Cas9 RNPs recognize each border of the target DNA nicking non-target strands, primers hybridize to the exposed non-target strands, SDA linearly replaces single -for and -rev strands, exponential SDA of target sequence, and amplicon quantification with biotin and Cy5-labeled PNA probes via magnetic pull-down and fluorescence measurements.	Highly specific and sensitive	Complex procedure; amplification step needed		200 bp	[107]
CRISPR-Cap	Escherichia coli	Cas9	For cleavage of target regions, a biotinylated sgRNA library and genomic DNA are mixed and incubated, cleaved CRISPR-DNA complexes are bound to streptavidin magnetic beads, and target DNA is released and sequenced.	Easy procedure; no mandatory amplification step	Reduced sequence length; high amount of sgRNAs needed	183.6-fold enrichment	13 kb	[108]
RGEN-R and RGEN-TdT	Mammalians	Cas9	Random fragmentation of genomic DNA (20–30 kb) and ddNTP treatment, Cas9-driven cleavage, and reparation with biotinylated adapters (RGEN-R) or with biotin-dUTP tail (RGEN-TdT), straptavidin-mediated isolation, PCR-free enrichment, and sequencing.	No amplification step needed; long sequences; low amount of sgRNAs needed	Highly dependent on the streptavidin purification step	5–60-fold enrichment	15 kb	[109]
	Human	Cas9	DNA digestion with EcoRI-HF and BamHI-HF and a sgRNA is designed adjacent to the region of interest. Digestion with Cas9 enables ligation with a capture adapter and SMRTbell molecules that contain the capture adapter are enriched on magnetic beads and prepared for SMRT PacBio sequencing.	No amplification step needed; long sequences	High amount of input DNA	up to 64,000-fold enrichment	1 kb	[110]
CISMR (CRISPR mediated isolation of specific megabase-sized regions of the genome	Yeast, Mouse	Cas9	CRISPR-mediated site-specific cleavage, isolation of megabase-sized DNA segment via PFGE, random fragmentation of DNA to 20 kb for Oxford Nanopore/PacBio long-read sequencing, or to 40 kb for fosmid clone library.	Long sequences	Complex procedure; not multiplexable; risk of yield loss during gel extraction	39–174-fold enrichment	up to 2.3 Mb	[111]
CATCH (Cas9-assisted targeting of chromosome segments)	Human	Cas9	Cells are embedded in an agarose gel-plug and lysed. Genomic DNA is Cas9-cleaved in the plug and the target DNA is separated by PFGE. The desired band is then excised from the gel, DNA is isolated, purified, and sequenced.	Long sequences	Complex procedure; not multiplexable; risk of yield loss during gel extraction; amplification step needed	237-fold enrichment	200 kb	[113]
nCATS (nanopore Cas9 Targeted-Sequencing)	Human	Cas9	DNA is fragmented, ends are dephosphorylated, and Cas9 -driven cleavage is performed. Nanopore sequencing adapters are ligated to new ends and fragments are sequenced.	Low amount of sgRNAs needed; no amplification step needed	Shorter sequences compared to other nCATS-like methods	10–300-fold enrichment	12.3–24.3 kb	[114]
	Mouse	Cas9	nCATS-like procedure with custom sequencing data analysis pipeline	Long sequences; low amount of sgRNAs needed; no amplification step needed	High amount of input DNA	400–700-fold enrichment	200 kb	[115]
FUDGE (FUsion Detection from Gene Enrichment)	Human	Cas9	nCATS-like procedure with custom sequencing data analysis pipeline	Low amount of sgRNAs needed; no amplification step needed	Shorter sequences compared to other nCATS-like methods	300–700-fold enrichment	up to 9.9 kb	[116]
ACME (Affinity-based Cas9 Mediated Enrichment)	Human	Cas9	nCATS-like procedure with a background reduction step	Long sequences; on-target to off-target ratio increase; no amplification step needed	Inability to sequence deeply targets >100 kb in size without coverage dropouts in the center	3–60-fold enrichment	200 kb	[117]
	Malus domestica	Cas9	nCATS-like procedure with custom sequencing data analysis pipeline	No amplification step needed; possible multiplexing	High quality input DNA needed	180× and 196× read depths	9.78 and 9.89 kb	[127]
	Prunus salicina	Cas9	nCATS-like procedure with custom sequencing data analysis pipeline	Long sequences; easy procedure; no previous knowledge of the DNA sequence is needed; no amplification step needed	Manual curation needed in case of de novo sequencing	11.9× read depth	194 Mb	[128]
	Triticale	Cas9	nCATS-like procedure with custom sequencing data analysis pipeline	Low sequencing depth for small loci in big genomes; no amplification step needed	No cost-effective if studying one or a few loci		3.4 kb, 5.1 kb and 3.6 kb	[118]
Cas9-tiling	Human; Rhesus macaques	Cas9	nCATS-like procedure on shorter sequences	Sequencing of small overlapping regions to avoid inaccuracies in polymorphism detections; no amplification step needed; further replacement of poor-quality assemblies in the reference genome	sgRNA design preferably on coding regions; high amount of sgRNAs needed depending on the entire sequence length	215–394-fold enrichment (human); 128–637-fold enrichment (macaques)	176 kb (human); 280 kb (macaques)	[124]
	Human	Cas9				19× to 238× target depth	28–100 kb	[125]
	Phaseolus vulgaris	Cas9				113.16-fold enrichment	250 kb	[126]
FLASH (Finding Low Abundance Sequences by Hybridization)	Pneumonia-causing gram+ bacteria; Plasmodium falciparum	Cas9	nCATS-like procedure with custom sequencing data analysis pipeline	Low amount of sgRNAs needed; low amount of input DNA	Amplification step needed; shorter sequences compared to other methods	>5000-fold enrichment		[120]
CANS (CRISPR-Associated Nanopore Sequencing)-NanoCasTE	Arabidopsis	Cas9	nCATS-like procedure with custom sequencing data analysis pipeline	Rapid procedure; no amplification step needed; high mappability of the reads	Shorter sequences compared to other methods	0.2× to 40× target depth	14–35 kb	[122]
s	Human	Cas9	nCATS-like procedure with gRNA specifically targeting 3’ end of the MEI sequences.	Low amount of sgRNAs needed; no amplification step needed; high sensitivity	Shorter sequences compared to other methods; possible recalcitrance of some chromosome locations	13.4× to 54× enrichment	14.9–32.3 kb	[121]
Negative enrichment	Human	Cas9	Cas9-driven cleavage of sequences flanking target region, treatment with exonucleases while Cas9 remains bound, protecting ends of target, purification from exonucleases and digested DNA, library preparation, and sequencing.	Easy procedure; low amount of sgRNAs needed; long sequences	Long persistence of Cas9 on the target	127 to 197-fold enrichment	36 kb	[97]
CAMP (CRISPR Associated Multiplexed PCR)	Human	Cas9	Cas9-driven cleavage at either side of the target locus. Universal UPS adapters are ligated for amplification. CAMP uses primers that have complementarity to the UPS adapter only, cCAMP uses chimeric primers that have complementarity to the UPS adapter and several bases of target DNA and cTRACE uses chimeric primers that have complementarity to the UPS adapter, several bases of target DNA, and specificity for a mutation.	High specificity; short DNA targeting; significant enrichment; multiplexable	Amplification step needed	2.6 × 10^4^ to 3.1 × 10^6^-fold enrichment	~100 kb	[119]
cCAMP (chimeric CRISPR Associated Multiplexed PCR)		Cas9		High specificity; significant enrichment; multiplexable	Amplification step needed	4.7 × 10^6^ to 2.1 × 10^8^-fold enrichment	~100 kb	
cTRACE (chimeric Targeting Rare Alleles with CRISPR-based Enrichment)		Cas9		Significant enrichment; flexibility in the location of the mutation in reference to a PAM site	Amplification step needed			
TRACE (Targeting Rare Alleles with CRISPR-based Enrichment)		Cas9	TRACE uses Cas9/sgRNA to protect targeted DNA from exonuclease, which digests off-target sequences; the protection provided by the Cas9 complex confers single base discrimination to protect a single base mutation while digesting the normal variant.	High specificity; No amplification step needed	Mutations must be in proximity to a PAM site; short sequences		up to 820 bp	
CRISPR-DS (CRISPR-Duplex Sequencing)	Human	Cas9	Cas9-driven cleavage of target sequence, targeted cutting of fragments containing coding exons using sgRNAs. Fragment size selection with beads. Double-stranded DNA fragmented and ligated with double-stranded DS adapters. Creation of single-strand consensus sequence (SSCS) reads and comparison to create a double-strand consensus sequence (DCS). Only mutations found in both SSCS reads are counted as true mutations in DCS reads.	Low amount of input DNA	Complex procedure; high quality input DNA needed; previous knowledge of the DNA target sequences needed	49,000-fold enrichment		[123]
	Human	dCas9	Target-bound dCas9 isolated through immunomagnetic precipitation, purification, and allele-specific qPCR.	Highly sensitive	Mutations must be in proximity to a PAM site	21-fold enrichment		[105]
CATE-seq (CRISPR-assisted targeted enrichment-sequencing)	Human	dCas9	DNA fragmentation, adapter ligation, target dCas9 binding, purification, and allele-specific PCR.	Highly specific and sensitive; low amount of sgRNAs needed	Complex procedure; short sequences	3700-fold enrichment	235 bp	[106]

## 8. Conclusions and Future Perspectives

Throughout this article, the authors have attempted to comprehensively report the numerous goals of non-editing approaches related to CRISPR/Cas-based system. Some of these specific applications have been developed as a tool to answer biological questions, for example, LiveFISH and gene regulation complexes, while others are set as purely diagnostic or functional tools such as SHERLOCK, the virus resistance engineering systems, or the ones that are used to facilitate sequencing. A couple (if not most) of them even have a place in both these categories, like CASFISH and its derivatives. The applications of CRISPR/Cas systems may be extremely diverse but the underlying mechanism remains the same in general: the Cas protein is sent to or recognizes a target of interest, specified by a purposely designed guide RNA, which triggers its activity or that of the effectors associated with it. The high specificity of the Cas-mediated sequence makes for the huge precision of the tools that are derived from it. The problem of off-target effects, inherent to the use of CRISPR/Cas proteins, however, remains; precautions must be taken and preliminary controls performed before making use of such techniques. Nevertheless, it has to be said that, when compared to equivalent techniques, the tools making use of CRISPR systems are often seen as less likely to have unspecific and undesired sequence binding [13,77]. We saw several times that using CRISPR/Cas-mediated tools produce clearer, more specific, and therefore more reliable results than not doing so while it is possible.

In addition to the goal of reviewing the various non-editing strategies developed and utilized in basic research up to this point, the authors also aimed to discuss the potential transferability of these strategies across different kingdoms. It is worth noting that while some of the applications mentioned were initially developed for plant systems, this scenario is more of an exception than a common occurrence. 

Indeed, most of the non-editing applications reviewed in this article have been developed on animal or human systems. There are several reasons as to why there are more non-editing applications of the CRISPR/Cas system in animals compared to plants: (i) historical emphasis: the initial development and application of CRISPR/Cas technology focused heavily on animal models, particularly mice, due to their well-established use in biomedical research. As a result, many of the early applications and optimizations of CRISPR/Cas were conducted in animal systems, leading to a greater body of research in this area; (ii) research priorities: the research priorities of funding agencies, academic institutions, and biotechnology companies may also play a role. Historically, there has been greater emphasis on biomedical research in animals due to its direct relevance to human health and disease, leading to more investment and resources allocated to CRISPR/Cas applications in animal models; (iii) technical challenges in plants: working with plants presents unique technical challenges compared to animals. Generating stable transgenic plant lines using CRISPR/Cas can be more time-consuming and labor-intensive due to factors such as the need for tissue culture, regeneration protocols, and genetic variability among plant species. These challenges have hindered the widespread adoption of CRISPR/Cas technology in plant research. Nonetheless, an increasing interest in the transferability of techniques from animal to plant kingdom is evident and most of the applications previously described could be successfully adapted and adopted in plant systems. In Table 5, a comparison and brief discussion of the availability of non-editing CRISPR-Cas-based tools in plants and animals is reported, with the general aim of triggering reasoning on the feasibility of technology transfer in different cases of study. In general, all those approaches that involve processing or treating cellular extracts, such as chromatin, purified nuclei, and genomic DNA/RNA, offer greater flexibility and adaptability across different biological systems as they are less dependent on specific physiological and structural characteristics of intact cells, tissues, and organs. Conversely, techniques that require manipulation within living tissues face greater hurdles in terms of compatibility and applicability across different organisms or experimental settings.

More generally, if the development of CRISPR/Cas systems-related technologies has opened a wide range of opportunities for the modification of genomic sequences, a deeper look reveals that this tool can also be used for non-editing purposes that are even more varied. Such understanding of the underlying genetic processes is a sine qua non to the overall goal of manipulating genes or regulatory sequences, may it be for therapeutic purposes in humans or to enhance the genetic traits of bacteria as well as domestic plants and animals. However, unlike for non-editing tools, interest in using such genome edition could rise and/or be democratized quicker in plants than in animals including humans, for the modification of the latter’s genome is still and should remain under strict ethical rules. Overall, these techniques facilitate both theoretical research, which is a crucial building block for later practical applications, and the practical applications in question. Understanding how genes function, how they are regulated, and how they interact with one another is essential to perform gene editing and regulatory sequence alteration in a meaningful and rigorous way.

To conclude, the ultimate objective of gene and regulatory sequence editing, whether for therapeutic use or genetic enhancement, necessitates a profound comprehension of gene functionality, regulation, and interactions. CRISPR-based strategies play a pivotal role in expanding this knowledge base, enabling both theoretical research and practical applications to thrive in the realm of genetic manipulation.

**Table 5:** Comparison of the availability of non-editing CRISPR-Cas-based tools in plants and in animals. Availability potential: still weak (∗); partial (∗∗); high (∗∗∗).

Category of Tools	Animals	Plants	Notes
Gene expression modulation	∗∗∗	∗	Most reported examples were developed on animals including humans. In plants, most of them are still lacking, presumably due to the time that it takes to import those approaches in vegetal systems. The inherent differences in the genome and cell structure between the two kingdoms could be an obstacle to the delivery and the functioning of the Cas system.
Study of transcription regulation	∗∗∗	∗	
Imaging/structural genomics	∗∗∗	∗∗	
Immunization against viruses and other pathogen	∗∗	∗∗∗	In the case of the techniques that aim at conferring immunity against viruses, it seems that the plant system is more in advance due to the high economic impact that such bioengineering could have on agriculture. Plus, in this case, the target of the Cas-mediated cleavage is no longer the endogenous genome of the plant cell but one of the viruses, whose structure is simpler. Application in animals must face stronger ethical considerations here, which may explain why there is more to find in vegetal systems when it comes to this field. On the contrary, the advances in medical care that would bring a better detection of pathogens account for the plethora of techniques that were developed in animals to assess the presence of pathogens; however, they are still mainly viral.
Detection of viruses and other pathogen	∗∗∗	∗∗	
NGS	∗∗∗	∗∗∗	The applicability of NGS-mediated techniques can be considered as being on the same level in both biological systems. Indeed, they are based on nucleic acids from cell extracts, which makes them independent from the biological properties of the system.

## Figures and Tables

**Table 1:** Cas9, Cas12a, and Cas13 at a glance. Abbreviations: SpCas9 = Cas9 from *Streptococcus pyogenes*; AsCas12a = Cas12a from *Acidaminococcus* spp.; LshCas13 = Cas13 from *Leptotrichia shahii*; HEPN = Higher eukaryotes and prokaryotes nucleotide-binding; crRNA = CRISPR RNA; tracrRNA = Trans-activating CRISPR RNA; PAM = Protospacer Adjacent Motif; ssRNA = Single Stranded RNA; LbCas12a = Cas12a from *Lachnospiraceae* spp.

Features	Cas9	Cas12a	Cas13
Other names		Cpf1	C2c2
Type of CRISPR/Cas system	Class 2 type II	Class 2 type V	Class 2 type VI
Size (amino acids)	1368 (SpCas9)	1307 (AaCas12a)	1389 (LshCas13a)
Nuclease domain	RuvC and HNH	RuvC	HEPN (×2)
Mutations inducing loss of function in nuclease domain.	D10A and H840A	-	D474A and D1046A
sgRNA components	crRNA and tracrRNA	crRNA	crRNA
sgRNA crRNA processing	tracrRNA-dependent	tracrRNA-independent	-
sgRNA protospacer length (nucleotides)	20 (minimum ensure DNA cleavage)	20	22–28
sgRNA total length (nucleotides)	>105	>42	>140
Targeted nuclei acid	dsDNA (can be induced to cleave ssRNA)	dsDNA, ssDNA (non-specific cleavage)	ssRNA
PAM sequence (5′-3′)	NGG (SpCas9)	T-rich, e.g., TTTV (AsCas12a, LbCas12a)	None
Cleavage	Blunt ended double-stranded break, 3 nucleotides before the PAM sequence. Each nuclease domain cleaves one strand.	PAM-distal dsDNA break with staggered 5′ and 3′ ends	Cleavage patterns depend on features of the target sequence (like accessibility) rather than the distance from the binding site. Single mismatches may be tolerated.
Other properties	-	Non-targeted ssDNA cleaving activity upon recognition of target sequence	Non-targeted ssRNA cleaving activity upon recognition of target sequence
Non-editing applications presented in this review	Modulation of gene expression and regulation Viral DNA targeting In situ DNA imaging New sequencing techniques	Modulation of gene expression and regulation Viral DNA targeting	In situ RNA imaging Viral RNA targeting Gene post-transcriptional regulation RNA detection techniques
References	[2,5,9,10]	[2,5,11]	[5,12,13,14]
